# Connectivity of Real-Time Video Counselling Versus Telephone Counselling for Smoking Cessation in Rural and Remote Areas: An Exploratory Study

**DOI:** 10.3390/ijerph17082891

**Published:** 2020-04-22

**Authors:** Judith Byaruhanga, Christine L. Paul, John Wiggers, Emma Byrnes, Aimee Mitchell, Christophe Lecathelinais, Flora Tzelepis

**Affiliations:** 1 School of Medicine and Public Health, University of Newcastle, University Drive, Callaghan, New South Wales 2308, Australia; chris.paul@newcastle.edu.au (C.L.P.); John.Wiggers@health.nsw.gov.au (J.W.); Emma.Byrnes@newcastle.edu.au (E.B.); Aimee.Mitchell@newcastle.edu.au (A.M.); flora.tzelepis@newcastle.edu.au (F.T.); 2 Hunter New England Population Health, Hunter New England Local Health District, Locked Mail Bag 10, Wallsend, New South Wales 2287, Australia; Christophe.Lecathelinais@health.nsw.gov.au; 3 Hunter Medical Research Institute, Locked bag 1000, New Lambton, New South Wales 2305, Australia

**Keywords:** connectivity, telemedicine, remote consultation, videoconferencing, telephone, rural

## Abstract

This study compared the connectivity of video sessions to telephone sessions delivered to smokers in rural areas and whether remoteness and video app (video only) were associated with the connectivity of video or telephone sessions. Participants were recruited into a randomised trial where two arms offered smoking cessation counselling via: (a) real-time video communication software (201 participants) or (b) telephone (229 participants). Participants were offered up to six video or telephone sessions and the connectivity of each session was recorded. A total of 456 video sessions and 606 telephone sessions were completed. There was adequate connectivity of the video intervention in terms of no echoing noise (97.8%), no loss of internet connection during the session (88.6%), no difficulty hearing the participant (88.4%) and no difficulty seeing the participant (87.5%). In more than 94% of telephone sessions, there was no echoing noise, no difficulty hearing the participant and no loss of telephone line connection. Video sessions had significantly greater odds of experiencing connectivity difficulties than telephone sessions in relation to connecting to the participant at the start (odds ratio, OR = 5.13, 95% confidence interval, CI 1.88–14.00), loss of connection during the session (OR = 11.84, 95% CI 4.80–29.22) and hearing the participant (OR = 2.53, 95% CI 1.41–4.55). There were no significant associations between remoteness and video app and connectivity difficulties in the video or telephone sessions. Real-time video sessions are a feasible option for smoking cessation providers to provide support in rural areas.

## 1. Introduction

The services routinely offered by quitlines for smoking cessation include telephone counselling (reactive and/or proactive) and written materials [1]. Telephone counselling is an accessible and flexible mode for delivering smoking cessation support [1,2,3] that has been shown to increase quitting success [4,5,6] and is cost effective [7]. The advantages of telephone services that assist people to quit include that: support can be tailored to individual needs; they are widely accessible; they eliminate the need to travel to access services; and they can reach rural and remote populations that may have limited access to in-person smoking cessation interventions [2].

Similar to telephone-based support, real-time video communication technology (e.g., Skype, FaceTime) has the potential to eliminate barriers to accessing face-to-face smoking cessation care [8,9,10]. In 2018, there were up to 3.9 billion internet users worldwide, representing more than half of the world’s population [11], with users being able to download video communication software for free [12]. In Australia, 86% of all households have access to the internet at home [13]. Eighty-eight percent of those living in major cities and 82.7% of those living in inner regional locations have internet access at home, as do 80.7% in outer regional locations and 77.1% in remote areas [13]. The advantages of real-time video communication technology include greater social support by replicating traditional physical face-to-face components [14], and raising motivation and encouragement [14,15]. Interpersonal interactions are crucial in persuading individuals into health promoting behaviours [14] and real-time video technology can facilitate interpersonal relations through responding to nonverbal cues and providing a social presence [16]. The opportunity to both see and hear the client during video-communication can result in a high level of engagement with the counsellor [17]. Three randomised trials conducted in the USA have compared video counselling to telephone counselling for smoking cessation [18,19,20]. In these studies, video counselling was offered to women living with HIV [18], Korean American women [19] and people living in rural areas [20]. However, none of these trials examined the connectivity of the video counselling sessions compared to telephone counselling sessions.

Quitlines have not implemented video counselling for smoking cessation as part of routine practice and a potential barrier may be the perceived connectivity of video communication technology such as the quality of the internet connection [8], video equipment or type of video application software making it difficult to use compared to other interventions [9,21]. According to the Australian Regional Telecommunications review report, video conferencing may be limited, particularly in areas serviced by satellite, and subsequently result in technical difficulties [22]. Therefore, a lack of good internet accessibility in some residential areas, such as rural and remote locations [22], may result in video communication delays, limited visibility and present a source of frustration and inconvenience for clients and health care providers [8,21,23,24,25].

No prior research has investigated the connectivity of real-time video counselling for smoking cessation in rural and remote locations. This study, therefore, aims to investigate:

(i) The connectivity of real-time video counselling sessions compared to telephone sessions delivered to smokers in rural and remote areas;
(ii) The factors (e.g., remoteness, video app (for video)) associated with connectivity of video sessions and telephone sessions.

## 2. Materials and Methods

### 2.1. Study Design

This study reports on the process measures of participants in a randomised smoking cessation trial, specifically, participants receiving smoking cessation counselling via either (1) real-time video counselling or (2) telephone counselling. A detailed description of the study design has been published elsewhere [26]. Briefly, smokers residing in rural and remote areas of New South Wales (NSW), Australia were randomly assigned to one of three conditions: (1) real-time video smoking cessation counselling; (2) telephone smoking cessation counselling; or (3) written smoking cessation materials (control). Given that the focus of this paper is on the connectivity of real-time video counselling compared to telephone counselling, data from only the former two conditions are reported. The study was conducted in accordance with the Declaration of Helsinki and the University of Newcastle Human Research Ethics Committee granted ethics approval (approval no. H-2016-0148). The trial is prospectively registered with the Australian New Zealand Clinical Trials Registry (ACTRN12617000514303).

#### 2.1.1. Setting and Participants

Between 25th May 2017 and 2nd October 2018, 655 participants were recruited from rural and remote locations of New South Wales (NSW), classified using the Accessibility and Remoteness Index of Australia (ARIA+) [27]. The ARIA+ is a geographic accessibility index that reflects the ease or difficulty people face accessing services in non–metropolitan Australia [27]. The different regions of rurality are established on the road distances needed to travel from the location to the service centres of various population sizes from a point to the nearest urban centres and localities in five separate population ranges [27]. The resulting index of geographic accessibility classification categorises locations as inner regional (> 0.2–≤ 2.4), outer regional (> 2.4–≤ 5.92), remote (> 5.92–10.53) and very remote (> 10.53) areas [27]. Participants were aged 18 years or older, used tobacco daily, had access to a mode of video communication (e.g., Skype, FaceTime), had internet access, telephone access and a current e-mail address, and resided in an inner or outer regional area or remote or very remote area of New South Wales (NSW) Australia [27].

#### 2.1.2. Procedure

Participants were recruited via traditional methods such as local newspapers, magazines, flyers and posters and via online strategies such as the study website, Facebook and Twitter [28]. Potential participants were asked to go to the project website that described the study, contained a detailed information statement and included the hyperlink to the online eligibility screening survey. After the online eligibility screening survey, eligible participants were automatically redirected to an online baseline survey, and at the end of the baseline survey a random number generator embedded into the computer software randomly allocated participants to condition.

### 2.2. Intervention Conditions

#### 2.2.1. Video Counselling Condition

Participants allocated to the video counselling condition received up to six video smoking cessation support sessions using the participant’s preferred form of video communication (e.g., Skype, FaceTime). The advisors used cognitive behaviour therapy [29] and motivational interviewing techniques [30] during the video sessions. The initial video session usually occurred within the first week of the participant enrolling into the trial.

Those participants that indicated readiness to quit within a month during the initial video session were offered five more counselling sessions on the quit date, and 3, 7, 14 and 30 days after the quit date. This evidence-based call schedule involves calls being scheduled close to the first two weeks following a quit attempt, which is the period where relapse often occurs [31].

Participants who indicated during the initial video session that they did not wish to quit within the next month were offered an additional three counselling calls at 2, 4 and 6 weeks after the initial call. The content of the video sessions included: assessment of smoking status and smoking history; identifying barriers to smoking cessation (e.g., drinking alcohol) and potential solutions; discussing effective smoking cessation strategies including both behavioural interventions and pharmacotherapies (e.g., Nicotine Replacement Therapy (NRT), bupropion, varenicline); and promoting self-efficacy and relapse prevention strategies to quit smoking. The mean duration of the video sessions was 19.18 min (standard deviation, SD 7.53).

#### 2.2.2. Telephone Counselling Condition

Telephone counselling participants received up to six smoking cessation telephone calls. The callback schedules, content and counselling techniques used in the telephone support calls were identical to those described for the video support sessions. The mean duration of the telephone calls was 16.08 min (SD 7.27).

### 2.3. Measures

#### 2.3.1. Connectivity of Video Counselling Sessions

After the completion of each video session, the smoking cessation advisor recorded in a database information related to the connectivity of the video session by answering the following questions: (a) *Did you have difficulty hearing the participant? (Yes/No).* (b) *Did you have difficulty seeing the participant? (Yes/No).* (c) *Was there an echoing noise during the video session? (Yes/No)* (d) *Did you lose internet connection during the video session? (Yes/No).* (e) *Did you find it difficult to connect to the participant at the start of the session? (Yes/No)* and (f) *Did you have difficulty operating the video application and equipment? (Yes/No).*

#### 2.3.2. Connectivity of Telephone Counselling Sessions

After the delivery of each telephone counselling session, the smoking cessation advisor recorded in a database information related to the connectivity of the telephone call by answering the following questions: (a) *Did you have difficulty hearing the participant? (Yes/No)*. (b) *Was there an echoing noise during the telephone session? (Yes/No)*. (c) *Did you lose telephone line connection during the telephone call? (Yes/No).* (d) *Did you find it difficult to connect to the participant at the start of the call? (Yes/No)* and (e) *Did you have difficulty operating the telephone equipment? (Yes/No).*

#### 2.3.3. Video Software Application Used (Video Group Only)

During the baseline survey, participants in the real-time video counselling condition were asked which video software application they preferred to use. The video software application was recorded by the counsellor for each video counselling session.

#### 2.3.4. Remoteness

Participants were asked to provide their residential postcode during the baseline survey, which was categorised using the Accessibility and Remoteness Index of Australia (ARIA+) into inner regional (> 0.2–≤ 2.4), outer regional (> 2.4–≤ 5.92), remote (> 5.92–≤ 10.53) or very remote (> 10.53) areas of New South Wales [27].

#### 2.3.5. Sociodemographic Characteristics

During the baseline survey, participant sociodemographic characteristics collected included age, gender, country of birth, Aboriginal or Torres Strait Islander origin, education, marital status, and occupational status.

### 2.4. Statistical Analysis

Statistical analysis was completed using the SAS software version 9.3 (SAS Institute Inc. Cary, NC, USA). Categorical data were described using frequencies and percentages.

For each connectivity issue assessed across both arms (hearing, echoing, operating, connecting, lost connection), video sessions were compared to telephone sessions by conducting Mixed Logistic Regression Models, to account for participant level clustering while adjusting for remoteness.

To examine the factors associated with each connectivity difficulty in the video sessions (seeing, hearing, echoing, operating, connecting, lost connection), the proportion of calls where the connectivity issue occurred was first assessed, and a threshold of 5% was set to determine whether there was enough variation in the data to warrant conducting further analysis. Multiple Logistic Mixed Regression models were used to determine whether the type of video app used, or the remoteness were associated with each eligible connectivity issue.

Similarly, to explore the factors associated with connectivity difficulties in the telephone sessions (hearing, echoing, operating, connecting, lost connection), the proportion of calls where each connectivity issue occurred was first assessed, and a threshold of 5% was set to reflect when there was some degree of variation in the data before further analysis was conducted. Multiple Logistic Mixed Regression models were used to determine whether remoteness was associated with each eligible connectivity issue.

## 3. Results

### 3.1. Participant Characteristics

Between 25 May 2017 and 2 October 2018, there was a total of 430 participants recruited into either the video counselling (*n* = 201) or telephone counselling (*n* = 229) arms of the randomised trial. Among the video counselling participants, the mean number of calls was 2.27 calls (SD = 2.44). Among the telephone counselling condition, the mean number of calls was 2.62 (SD = 2.35). There was no significant difference in the mean number of calls between the video and telephone counselling conditions. Table 1 describes the participant characteristics for each of these conditions. There were no significant between-group differences for any characteristic at baseline.

### 3.2. Connectivity of Video Sessions

Overall, 456 video counselling sessions were completed. Of the 456 counselling sessions, 236 (51.8%) were delivered via Facebook Messenger, 157 (34.4%) via FaceTime, 58 (12.7%) via Skype and 5 (1.1%) via Google Hangouts. For 99.6% (454/456) of video sessions, the advisors had no difficulty operating the video equipment, 97.8% (446/456) of video sessions had no echoing noise, and in 94.5% (431/456) there was no difficulty connecting with the participant at the start of the session. In 88.6% (404/456) of the video sessions, there was no loss of internet connection during the session, and in 88.4% (403/456) there was no difficulty hearing the participant, while in 87.5% (399/456) of the video sessions there was no difficulty seeing the participant (Table 2).

### 3.3. Connectivity of Telephone Calls

Overall, 606 telephone counselling calls were completed. Table 2 outlines the connectivity of these calls. The advisors had no difficulty using the telephone equipment for all telephone counselling calls. For the vast majority of telephone calls, there was no echoing noise during the session (99.8%, 605/606), no difficulty connecting to the participant at the start of the call (99.0%, 600/606) and no loss of telephone line connection during the telephone call (98.8%, 599/606). In 94.6% (573/606) of the telephone calls there was no difficulty hearing the participant.

### 3.4. Comparison of Connectivity between Telephone and Video Sessions

After adjusting for remoteness and participant level clustering, the video sessions had significantly greater odds, compared to the telephone sessions, of difficulty hearing the participant (OR 2.53, 95% CI 1.41–4.55), loss of connection during the session (OR 11.84, 95% CI 4.80–29.22), and difficulty connecting at the start of the session (OR 5.13, 95% CI 1.88–14.00) (Table 2).

### 3.5. Factors Associated with Connectivity Issues during the Video Sessions

Where a particular connectivity difficulty was reported for >5% of the video sessions, we explored the characteristics associated with that connectivity measure. For the video sessions, there were no characteristics significantly associated with (a) difficulty seeing the participant, (b) difficulty connecting to the participant at the start of the video session, (c) loss of internet connection during video session (Table 3) and difficulty hearing the participant (Table 4).

### 3.6. Factors Associated with Connectivity Issues during the Telephone Calls

Where connectivity difficulties were reported for >5% of the telephone calls, we explored the characteristics associated with each of those connectivity measures. As shown in Table 4, there were no factors significantly associated with difficulty hearing the participant during the telephone calls.

## 4. Discussion

This is the first study to examine the connectivity of real-time video smoking cessation sessions and telephone calls in rural and remote locations. The findings demonstrated that real-time video counselling sessions were feasible for most rural smokers with only minimal connectivity problems.

Poor internet connection has been cited as a challenge in using video communication technology in Australia and internationally [8] and is considered a potential limitation for delivering interventions over the internet [8]. This study, however, found that for the video sessions delivered to rural residents there were no connectivity issues for the vast majority of the video sessions, suggesting that providing smoking cessation support via video communication software is feasible. When connectivity issues were identified for the video sessions, the concerns most commonly related to difficulty seeing the participant (12.5%), difficulty hearing the participant (11.6%) and loss of internet connection during the video session (11.4%). In Australia, 17.3% of those living in inner regional locations, 19.3% in outer regional locations and 22.9% in remote areas do not have internet access at home [13], which reduces the reach of video counselling in these areas. In regard to the telephone calls, there were few instances of connectivity difficulties in terms of difficulty hearing the participant (5.4%), loss of telephone line connection during the call (1.2%), difficulty connecting at the start of the call (1%) and echoing (0.2%). These findings provide further support for delivering smoking cessation care via telephone in rural locations which is the approach used by quitline providers as part of their routine practices [1,32,33].

Video sessions had significantly greater odds of connectivity difficulties than telephone sessions in relation to connecting to the participant at the start of the session, losing connection during the session and difficulty hearing the participant. This suggests that the delivery of smoking cessation counselling via telephone or video should operate on a flexible model, where connectivity is a key criterium. For people in rural areas who cannot easily access existing face to face services due to associated travel costs [34,35], both real-time video communication and telephone interventions are feasible modes for the delivery of smoking cessation support. Counselling via video or telephone is an option for any individual where connectivity allows.

Understanding whether any factors are associated with the connectivity of video sessions or telephone calls, could help to identify for which subgroups improved technological infrastructure is needed for the delivery of such interventions. However, in this study there were no factors associated with connectivity difficulties for the video sessions and for the telephone calls. This suggests that the delivery of real-time video counselling and telephone counselling for smoking cessation is feasible in terms of connectivity, irrespective of rural location and video app (video sessions only). Given that there were very limited data collected in remote locations and none in very remote locations, this study is unable to draw conclusions about connectivity difficulties in remote and very remote locations for either video communication or telephone.

The study had a number of limitations. First, there was a very small number of video or telephone sessions delivered to remote areas and none to very remote locations, and therefore the findings reported predominately reflect the connectivity difficulties experienced in rural areas. Second, data were not collected about the type of the device the participant used (smartphone/tablet or computer), internet provider (e.g., Optus, Telstra) their internet connection (mobile internet, broadband or NBN) or subscription option (prepaid or post-paid), and therefore we were unable to determine whether these factors were associated with connectivity difficulties during the video sessions. Future research should collect such information to assess whether these factors are associated with connectivity difficulties. Third, participants were recruited via online and traditional recruitment methods and therefore there may be self-selection bias. It is, however, important to note that the recruitment methods used in this study are the same as those used by real-world quitlines to enroll clients into their services. Therefore, our study recruited people seeking treatment, which is the sub-group that quitlines and other smoking cessation services target to use their services. Fourth, this study examined connectivity difficulties in video or telephone sessions in rural and remote areas of New South Wales, Australia, and there may be limited generalisability of the findings to other geographical locations. Fifth, the connectivity of the video and telephone sessions were only recorded from the service provider’s perspective and no information about the connectivity of each session was collected from the participant’s perspective.

## 5. Conclusions

The connectivity of real-time video counselling and telephone counselling for smoking cessation was sufficiently achievable in rural locations. There were no factors identified in this study that were associated with connectivity difficulties in rural locations for either video counselling or telephone counselling. Quitlines and other smoking cessation providers could consider integrating a real-time video counselling option as part of their services. Further research is needed to examine the connectivity and feasibility of video sessions in remote and very remote areas.

## Figures and Tables

**Table 1:** Participant characteristics in video and telephone conditions.

Characteristics	Categories	Video	Telephone
		*N* = 201	*N* = 229
		*n* (%)	*n* (%)
Gender			
	Female	158 (78.6%)	174 (76.0%)
	Male	43 (21.4%)	55 (24.0%)
Education			
	Year 10 or less	51 (25.6%)	71 (31.0%)
	HSC ^a^/Year 12 or TAFE ^b^	92 (46.2%)	116 (50.7%)
	University or tertiary	56 (28.1%)	42 (18.3%)
Marital status			
	With Partner	112 (55.7%)	134 (58.5%)
	Without partner	89 (44.3%)	95 (41.5%)
Employment			
	Employed full/casual/part time	132 (65.7%)	138 (60.3%)
	Not Employed	69 (34.3%)	91 (39.7%)
Aboriginal or Torres Strait Islander			
	Yes	13 (6.5%)	23 (10.0%)
	No	188 (93.5%)	206 (90.0%)
Australian born			
	No	26 (12.9%)	28 (12.2%)
	Yes	175 (87.1%)	201 (87.8%)
			
Remoteness	Inner Regional Australia	149 (74.1%)	167 (73.6%)
	Outer Regional Australia	49 (24.4%)	57 (25.1%)
	Remote Australia	3 (1.5%)	3 (1.3%)

*N* = total number; *n* = number of participants; ^a^ HSC: Higher School Certificate. ^b^ TAFE: Technical and Further Education.

**Table 2:** Connectivity of video sessions and telephone sessions in rural and remote locations ^a^.

	Telephone *n* = Number of Sessions		Video *n* = Number of Sessions		Between Group Analysis	
	Yes *n* (%)	No *n* (%)	Yes *n* (%)	No *n* (%)	Odds Ratio (95% CIs) (Video vs. Telephone)	*p*
Did you have difficulty seeing the participant?	N/A	N/A	57 (12.5)	399 (87.5)	^b^	
Was there an echoing noise during the session?	1 (0.2)	605 (99.8)	10 (2.2)	446 (97.8)	^c^	
Did you have difficulty operating the video app/telephone equipment?	0	606 (100)	2 (0.4)	454 (99.6)	^c^	
Did you find it difficult to connect to the participant at the start?	6 (1)	600 (99.0)	25 (5.5)	431 (94.5)	5.13 (1.88–14.00)	0.001
Did you lose connection during telephone/video call?	7 (1.2)	599 (98.8)	52 (11.4)	404 (88.6)	11.84 (4.80–29.22)	<0.0001
Did you have difficulty hearing the participant?	33 (5.4)	573 (94.6)	53 (11.6)	403 (88.4)	2.53 (1.41–4.55)	0.002

^a^ Adjusted for remoteness and participant level clustering, ^b^ OR not applicable as no between group comparisons ^c^ OR could not be estimated due to small *n* or *n* = 0.

**Table 3:** Factors associated with connectivity issues during the video sessions ^a^.

	Difficulty Seeing During Video Call			Difficulty Connecting at Start of Video Call			Loss of Internet Connection during Video Call			Difficulty Hearing during Video Call		
	*n* (%)	OR (95% CI)	*p*	*n* (%)	OR (95% CI)	*p*	*n* (%)	OR (95% CI)	*p*	*n* (%)	OR (95% CI)	*p*
Video App			0.88			1			1			0.33
Facetime (*N* = 157)	16 (10.2)	0.61 (0.15–2.52)		7 (4.5)	1.25 (0.20–7.91)		20 (12.7)	1.15 (0.33–4.08)		12 (7.6)	0.94 (0.20–4.36)	
Facebook (*N* = 236)	34 (14.4)	0.84 (0.23–3.17)		15 (6.4)	1.16 (0.21–6.58)		26 (11.0)	1.09 (0.33–3.61)		37 (15.7)	2.2 (0.52–8.89)	
Google Hangouts (N = 5)	0 (0)	^b^		0 (0)	^b^		0 (0)	^b^		0 (0)	^b^	
Skype (*N* = 58)	7 (12.1)	Referent		3 (5.2)	Referent		6 (10.3)	Referent		4 (6.9)	Referent	
Remoteness			0.98			0.14			0.18			0.85
Inner regional (*N* = 341)	45 (13.2)	1.01 (0.36–2.82)		23 (6.7)	3.69 (0.66–20.84)		35 (10.3)	0.56 (0.24–1.31)		35(10.3)	0.91 (0.34–2.44)	
Outer regional and remote (*N* = 115)	12 (10.4)	Referent		2 (1.7)	Referent		17 (14.8)	Referent		17 (14.8)	Referent	

^a^ Adjusted for participant level clustering. ^b^ *n* = 0 and OR could not be estimated.

**Table 4:** Factors associated with connectivity issues during the telephone calls ^a^.

		Difficulty Hearing the Participant OR (95% CI)	*p*
**Remoteness**	***n* (%)**		0.67
Inner regional (*N* = 432)	21(4.9)	0.81(0.32–2.10)	
Outer Regional/Remote (*N* = 168)	11(6.6)	Referent	

^a^ Adjusted for participant level clustering.
